# Interactions involved in the adsorption of ethylene glycol and 2-hydroxyethoxide on the Au(111) surface: a Density Functional Theory study

**DOI:** 10.1007/s00894-024-06187-6

**Published:** 2024-11-12

**Authors:** Joana Avelar, Raymundo Hernández-Esparza, Jorge Garza, Rubicelia Vargas

**Affiliations:** 1 Departamento de Química, División de Ciencias Básicas e Ingeniería, Universidad Autónoma Metropolitana-Iztapalapa, San Rafael Atlixco 186, Col, Vicentina, 09340 Iztapalapa, Ciudad de México México; 2 Leadership Computing Facility, Argonne National Laboratory, 9700 South Cass Avenue, Lemont, IL 60439 USA

**Keywords:** Au surface, Ethylene glycol degradation, DFT, QTAIM

## Abstract

**Context:** The monolayers of ethylene glycol and 2-hydroxyethoxide on gold surfaces have been used in hybrid materials as biosensors. In this article, the adsorption of ethylene glycol and 2-hydroxyethoxide on the Au(111) surface was analyzed. For the first system, ethylene glycol on Au(111), there are Au$\cdot \cdot \cdot$O and Au$\cdot \cdot \cdot$H interactions. To the best of our knowledge, the Au$\cdot \cdot \cdot$H interaction has been overlooked until now. However, in this work, there is strong evidence that this interaction is important to stabilize the system. For the second system, the atomic interactions mentioned previously are also predicted, although there is an additional interaction between 2-hydroxyethoxide molecules. Such an interaction induces the link -O-H-O-, with high values of the electron density at the critical points of the corresponding bond path of the O-H interaction. These links suggest the forming of ethylene glycol chains. **Methods:** The calculations were performed using two exchange-correlation functionals: BEEF-vdW and C09$_{x}$-vdW; both functionals incorporate dispersion effects within the Kohn-Sham approach in Density Functional Theory as implemented in GPAW code and ASE computational packages. The contacts between the molecules considered in this article and the Au(111) surface were analyzed through the Quantum Theory of Atoms in Molecules implemented in GPUAM code.

## Introduction

Ethylene glycol is a diol commonly used as a coolant, antifreeze, and de-icing agent that has gained significant attention due to its potential applications in various fields, including biomedicine and catalysis [1–5]. The modification of metallic surfaces with ethylene glycol improves the surface properties for some chemical processes. In specific, gold surfaces have been explored as a platform for the functionalization and application of ethylene glycol [6–8].

Ethylene glycol is a versatile compound; it has been employed in various contexts, including the synthesis of gold nanoparticles and the development of hybrid materials. Gold nanoparticles (AuNPs) synthesized using ethylene glycol as a reducing agent have shown great potential in various applications, such as drug delivery and cellular imaging, due to their stability and biocompatibility [9–13].

**Fig. 1:**
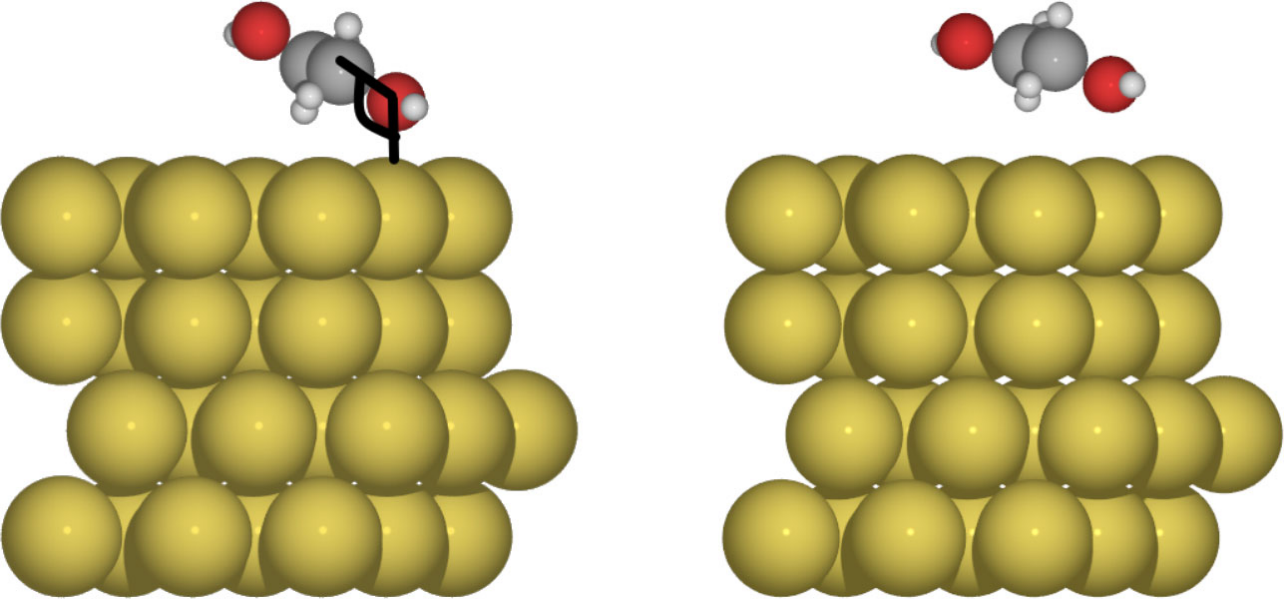
Ethylene glycol adsorption on Au(111) for C09$_{x}$-vdW (left side) and BEEF-vdW (right side) exchange-correlation functionals

The hybrid materials have shown promise in enhancing the performance of biosensors by reducing nonspecific binding and improving the stability of the sensing elements. One advantage of using ethylene glycol is its ability to form a monolayer on gold surfaces, which effectively shields the nanoparticles from nonspecific interactions with biological molecules. This property is particularly important in biosensors, where the interaction between the sensing element and the target analyte is critical for accurate detection. Furthermore, the use of 2-hydroxyethoxide, a derivative of ethylene glycol, has also been explored in the context of biosensors. This compound has been shown to enhance the stability and biocompatibility of gold nanoparticles, making them suitable for use in biological systems [6, 14]. The integration of these materials into biosensors has led to significant improvements in their performance. For instance, the use of gold nanoparticles loaded with ethylene glycol has been demonstrated to enhance the sensitivity and detection limit of hydrogen peroxide sensors. Similarly, the use of hybrid materials composed of gold nanoparticles and 2-hydroxyethoxide has been shown to improve the stability and biocompatibility of biosensors.

On the other hand, the dehydrogenation process of polyols on metallic surfaces begins with the adsorption of oxygen atoms, followed by the cleavage of the O-H bond to form an alkoxide intermediate and, ultimately, the breaking of a C-H bond. Previous research suggests that the O-H bond cleavage is the bottleneck step, specifically on Pt surface [15]. However, other studies indicate that the final C-H bond cleavage may be the determining step [16, 17]. Additionally, intermolecular interactions between reactants and products on the catalytic surface can influence reaction energetics, significantly impacting the oxidation process [18].

**Table 1:** Adsorption energies (E$_{ads}$), adsorption angles ($\alpha _{ads}$), and distance O-Au of ethylene glycol on Au(111)

Property	E$_{ads}$ (eV)	$\alpha _{ads}$ (degrees)	d$_{O-Au}$ (Å)
C09$_{x}$-vdW	$-$0.64	43.4	2.67
BEEF-vdW	$-$0.61	48.7	3.04

It is recognized that intermolecular interactions, also known as non-covalent interactions, play a relevant role in the stability of many chemical systems such as biomolecules, molecular crystals, and polymers [19]. Extensive experimental and theoretical studies have consistently demonstrated the significance of these interactions. However, non-covalent interactions at the interface between molecules and surfaces have not been extensively studied or characterized, despite their significant role in stabilizing these systems and influencing the properties of adsorbed molecules or modified surfaces.

Density Functional Theory (DFT) is the cornerstone to describing the electronic structure of atoms, molecules, or solids. The implementation of DFT methods is now an important piece of computational methods. It is widely recognized that within the Kohn-Sham approach, the choice of exchange-correlation functional significantly influences results. When studying intermolecular interactions, it is essential to employ either dispersion correction methods or exchange-correlation functionals that inherently account for these interactions, such as van der Waals functionals (vdW-DFT) [20]. While these types of exchange-correlation functionals have been proven to yield accurate results for bulk properties, energy surfaces, adsorption energies, and other relevant properties related to periodic systems, they are not widely utilized despite their effectiveness in these applications.

In this study, we investigate the adsorption of ethylene glycol and 2-hydroxyethoxide on the gold surface (111) to characterize their non-covalent interactions and monolayer formation using van der Waals exchange-correlation functionals. Two distinct vdW-DFT exchange-correlation functionals are employed in this article. To analyze the intermolecular interactions between molecules and the gold surface, we utilize the Quantum Theory of Atoms in Molecules (QTAIM) [21], with the electron density obtained from vdW-DFT computations.

**Fig. 2:**
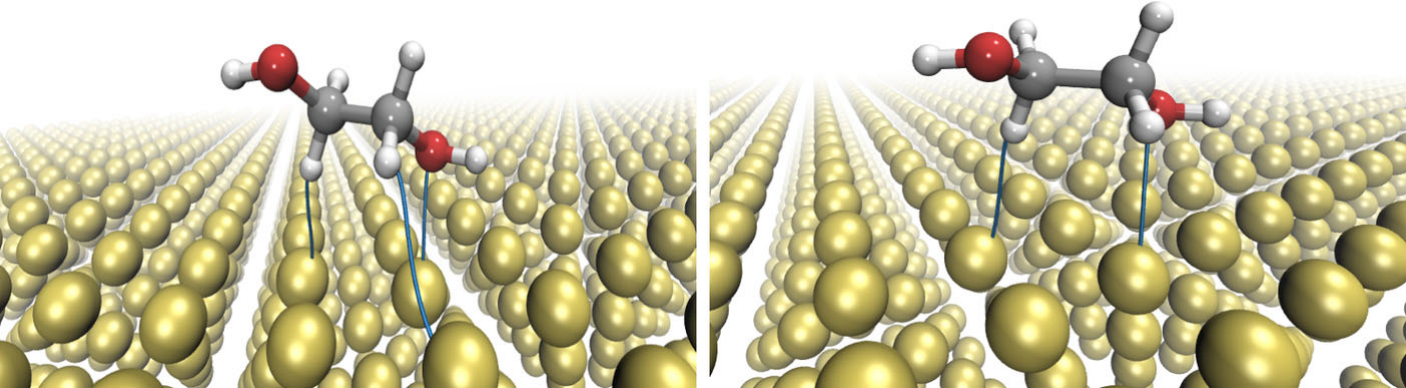
Bond paths for ethylene glycol adsorption on Au(111) obtained by C09$_{x}$-vdW (left side) and BEEF-vdW (right side) exchange-correlation functionals

In addition to QTAIM, non-covalent interactions (NCI) analysis was employed in this study. The NCI method utilizes electron density and its reduced gradient to visualize possible non-covalent interactions [22–24]. Originally designed for finite systems, this approach has been extended by various groups and implemented in computational algorithms for systems under periodic boundary conditions [23, 25, 26]. Our group specifically developed a code tailored for this purpose [27, 28]. Without a doubt, both QTAIM and NCI are valuable tools for identifying interactions between adsorbates and surfaces.

## Methodology

Calculations were performed by using DFT as implemented in GPAW code [29–31] and ASE computational packages. The projector-augmented wave method (PAW)[32, 33] described the atomic cores. It is an all-electron full-potential method within the frozen core approximation, including scalar relativistic effects. The exchange-correlation functionals used are C09-vdW [34] and BEEF-vdW [35] to consider van der Waals effects. These functionals have been tested for bulk and surface properties of transition metals [36]. A plane-wave cutoff of 450 eV was used while the Brillouin zone was sampling as a 4$\times$4$\times$1 Monkhorst-Pack [37] grid.

The surface periodic slab was modeled by a (3$\times$3) four-atomic-layer slab and a vacuum of 20 Å, allowing up to three molecules could be fitted within this region. During the geometry optimization, the two top layers, together with adsorbate, were allowed to relax, while the bottom layers were frozen at the bulk optimized positions. The lattice parameters calculated for bulk Au were 4.14 and 4.25 Å for C09-vdW and BEEF-vdW, respectively, in good agreement with the experimental value (4.07 Å) [38]. The total energy of isolated molecules was calculated from the molecule centered in a unit cell of 16$\times$16$\times$16 Å. These calculations were carried out at the $\Gamma$ point, and spin-polarization was used when it was required.

**Table 2:** Contacts; bond distances, d$_{A-B}$; electronic density in the bond critical point, $\rho _{BCP}$; and Laplacian of the density in the BCP, $\nabla ^{2}\rho _{BCP}$, for HOCH$_{2}$CH$_{2}$OH/Au(111) with C09$_{x}$-vdW and BEEF-vdW exchange-correlation functionals

Interaction	d$_{A-B}$ (Å)	$\rho _{BCP}$ (u.a.)	$\nabla ^{2}\rho _{BCP}$ (u.a.)
C09$_{x}$-vdW			
O$\cdot \cdot \cdot$Au	2.67	0.032	0.205
H$\cdot \cdot \cdot$Au	2.53	0.013	0.028
H$\cdot \cdot \cdot$Au	3.02	0.007	0.016
BEEF-vdW			
O$\cdot \cdot \cdot$Au	3.00	0.014	0.058
H$\cdot \cdot \cdot$Au	2.91	0.008	0.020

The adsorption energies were evaluated as the difference between the energy of the molecule adsorbed on Au(111) surface ($E_{Mol/Au}$), the molecule energy ($E_{Mol}$), and the surface energy ($E_{Au}$) as follows:

1 $$\begin{aligned} E_{ads}=E_{Mol/Au}-E_{Mol}-E_{Au} \end{aligned}$$

In order to analyze the lateral intermolecular interactions between reactants and products, the coverage effect of HOCH$_{2}$CH$_{2}$O on Au(111) was evaluated according the definition of the coverage ($\theta$), which is defined as the ratio of number of molecules adsorbed on the surface ($n_{mol}$) and the number of metallic atoms on the surface ($n_{atom-surf}$).

2 $$\begin{aligned} \theta = \frac{n_{Mol}}{n_{atom-surf}} \end{aligned}$$

The adsorption energy of these systems was calculated as

3 $$\begin{aligned} E_{ads} = E_{n-Mol/surf} - nE_{Mol} - E_{surf} \end{aligned}$$

where $E_{n-Mol/surf}$ is the energy of *n* molecules adsorbed on the metallic surface, and $E_{Mol}$ and $E_{surf}$ correspond to the isolated molecule energy and the surface energy, respectively.

To characterize the interactions present in the interphase of these systems, we used QTAIM and NCI methods as they are implemented in *Graphics Processing Units for Atoms in Molecules (GPUAM)* code [27, 28]. The non-covalent interactions are analyzed with the value of the electron density on the bond critical point ($\rho _{BCP}$) and the Laplacian ($\nabla ^2\rho _{BCP}$) at this point. We also use the usual isosurface color of the NCI; the vdW attractive interactions are shown in green, while blue and red indicate the attractive and strained interactions, respectively.

**Fig. 3:**
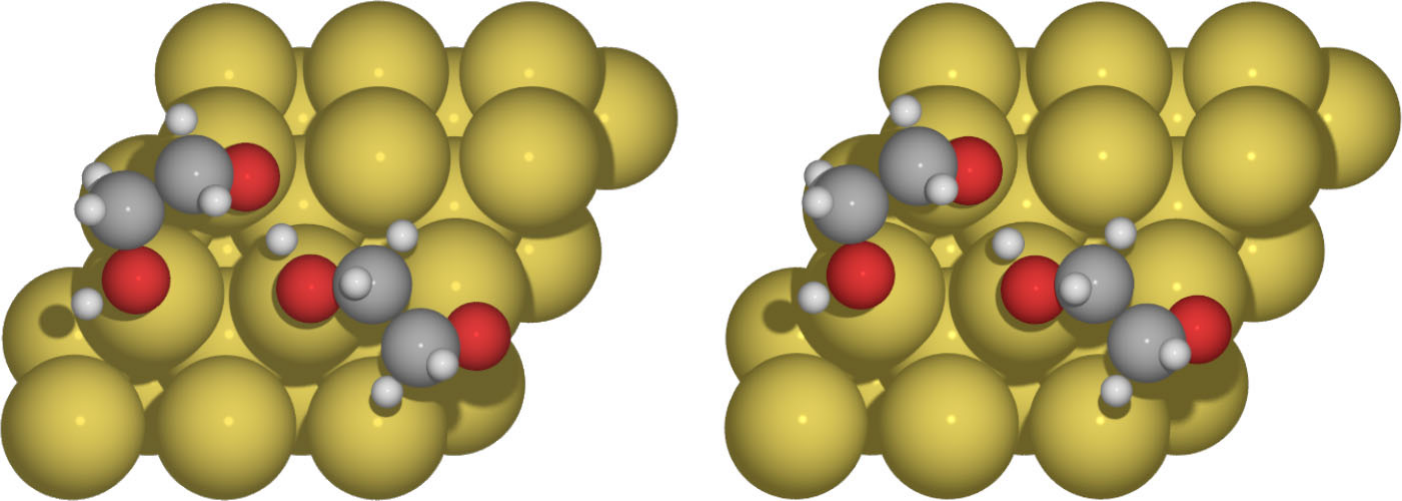
Optimized structures for $\theta$=2/9 of HOCH$_{2}$CH$_{2}$O on Au(111) with C09$_{x}$-vdW (left side) and BEEF-vdW (right side) exchange-correlation functionals

**Fig. 4:**
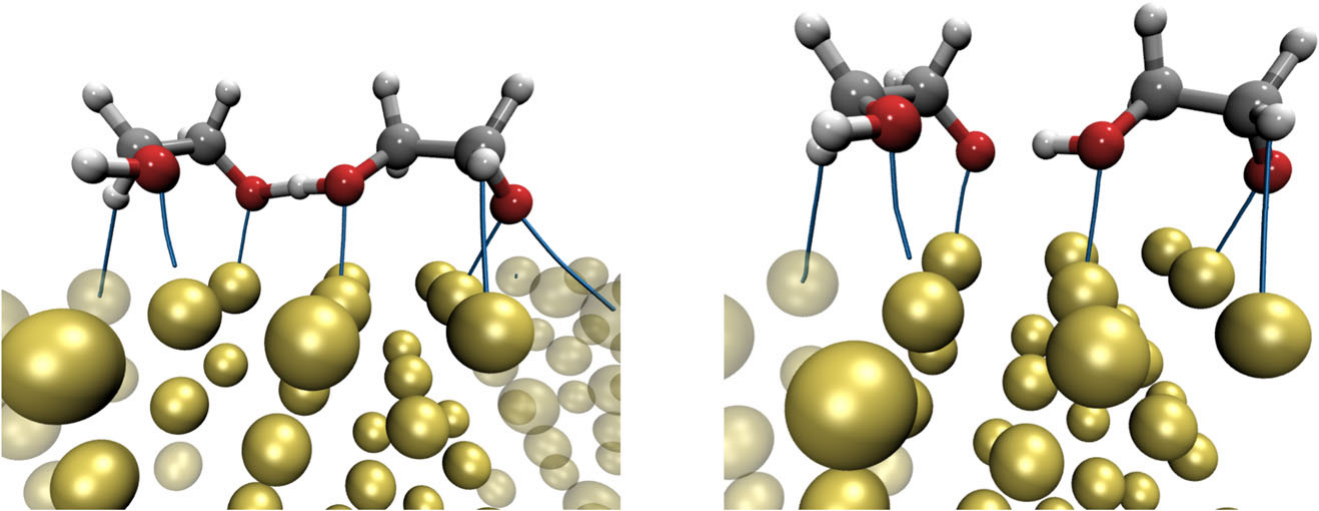
Bond paths for $\theta$=2/9 of HOCH$_{2}$CH$_{2}$O on Au(111) C09$_{x}$-vdW (left side) and BEEF-vdW (right side) exchange-correlation functionals

## Results and discussion

### Ethylene glycol on the Au(111) surface

With both exchange-correlation functionals considered in this article, the adsorption of ethylene glycol on Au(111) occurs, as expected, by the interaction of an oxygen atom with Au atoms from the surface on top sites. The molecule is adsorbed with a tilt angle $\alpha$ (see Fig. 1) equal to 43.4$^{\circ }$ for C09$_{x}$-vdW and 48.7$^{\circ }$ for BEEF-vdW, while bond distances O-Au are within 2.5$-$3.2 Å range, which is consistent with vdW interactions previously reported [39]. These results, together with adsorption energies, are reported in Table 1. There is evidence of an attractive interaction between ethylene glycol and the Au(111) surface since both exchange-correlation functionals predict adsorption energies around $-$0.6 eV. For reference, let us remember that the water dimer exhibits a non-covalent interaction around $-$0.2 eV [40].

We estimated the adsorption of ethylene glycol on gold using exchange-correlation functionals with and without dispersion corrections. Exchange-correlation functionals without a dispersion correction predict quite small adsorption energies compared to those obtained by BEEF-vdW and C09$_{x}$-vdW; for this reason, exchange-correlation without dispersion corrections are not considered in this article.

Additionally, we used QTAIM theory to analyze the interactions present in the systems studied in this work. This theory predicts H$\cdot \cdot \cdot$Au and O$\cdot \cdot \cdot$Au interactions for the adsorption of ethylene glycol on the Au(111) surface with C09*x*-vdW and BEEF-vdW xc functionals (see Fig. 2). The positive $\nabla ^{2}\rho _{BCP}$ and $\rho _{BCP}$ values reported in Table 2 indicate that these interactions can be classified as non-covalent interactions. This information, in conjunction with interaction energies, corroborates that the adsorption of ethylene glycol on Au(111) corresponds to a physisorption process.

These results are in good agreement with experimental observations about oxygen interaction with Au [41]. While the interaction H$\cdot \cdot \cdot$Au has not been identified before, in this study, this interaction appears with both functionals as it is shown in Fig. 2. This interaction cannot be underestimated since the distance H$\cdot \cdot \cdot$Au is similar to that observed for O$\cdot \cdot \cdot$Au. Additionally, the electron density evaluated at the bond critical point ($\rho _{BCP}$) of the H$\cdot \cdot \cdot$Au interaction represents more than 40% of the same property for the O$\cdot \cdot \cdot$Au contact. Thus, there is strong evidence that this interaction assists the cleavage of the O-H bond.

The C09$_{x}$-vdW exchange-correlation functional predicts shorter and stronger interactions than BEEF-vdW; besides this exchange-correlation functional, it predicts an additional H$\cdot \cdot \cdot$Au interaction (see Table 2). Although such an interaction is quite weak, since the distance H$\cdot \cdot \cdot$Au is large and its corresponding $\rho _{BCP}$ is small.

### Dehydrogenated ethylene glycol on Au(111) surface

It is well known that the adsorption of ethylene glycol on metallic surfaces causes its decomposition. In general, the rupture of C-C/C-O bonds is more difficult than breaking C-H/O-H bonds. It is expected that the first bond breaking of ethylene glycol occurs in the bonds O-H or C-H. The interaction between dehydrogenated ethylene glycol and Au(111) surface is crucial to understanding ethylene glycol decomposition. Several authors have reported experiments showing the formation of monolayers of dehydrogenated ethylene glycol on the Au(111) surface [42, 43]. Results indicate strong adsorption of HOCH$_{2}$CH$_{2}$O on gold surface. For this reason, the adsorption effect of two and three molecules of HOCH$_{2}$CH$_{2}$O on Au(111) is considered in this article. Such a number of molecules correspond to 2/9 and 3/9 ML coverages.

**Table 3:** Contacts; bond distances, d$_{A-B}$; electronic density in the bond critical point, $\rho _{BCP}$; and Laplacian of the density in the BCP, $\nabla ^{2}\rho _{BCP}$ for two molecules of HOCH$_{2}$CH$_{2}$O adsorb on Au(111) with C09$_{x}$-vdW and BEEF-vdW for $\theta$=2/9 ML

Interaction	d$_{A-B}$ (Å)	$\rho _{BCP}$ (u.a.)	$\nabla ^{2}\rho _{BCP}$ (u.a.)
C09$_{x}$-vdW			
O$\cdot \cdot \cdot$Au	2.22	0.080	0.212
O$\cdot \cdot \cdot$Au	2.22	0.080	0.202
O$\cdot \cdot \cdot$Au	2.30	0.067	0.153
O$\cdot \cdot \cdot$Au	2.44	0.051	0.084
O$\cdot \cdot \cdot$Au	2.81	0.023	0.092
H$\cdot \cdot \cdot$Au	2.19	0.037	0.078
H$\cdot \cdot \cdot$Au	2.63	0.015	0.036
H-O	1.04	0.282	$-$1.698
H-O	1.09	0.243	$-$0.897
H$\cdot \cdot \cdot$O	1.38	0.114	$-$0.126
H$\cdot \cdot \cdot$O	1.51	0.080	$-$0.018
BEEF-vdW			
O$\cdot \cdot \cdot$Au	2.25	0.075	0.113
O$\cdot \cdot \cdot$Au	2.26	0.072	0.184
O$\cdot \cdot \cdot$Au	2.54	0.038	0.105
O$\cdot \cdot \cdot$Au	2.74	0.025	0.067
H$\cdot \cdot \cdot$Au	2.43	0.022	0.041
H$\cdot \cdot \cdot$Au	3.02	0.007	0.021
H-O	1.02	0.328	$-$1.789
H-O	1.01	0.317	$-$3.728
H$\cdot \cdot \cdot$O	1.59	0.065	0.069
H$\cdot \cdot \cdot$O	1.63	0.059	$-$0.043

**Fig. 5:**
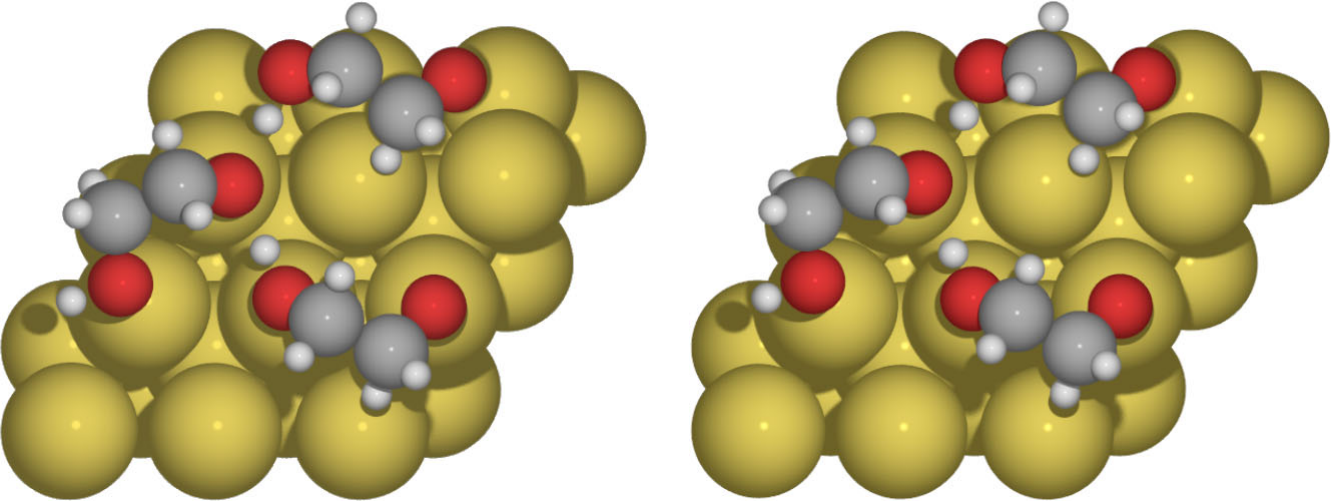
Optimized structures for $\theta$=3/9 of HOCH$_{2}$CH$_{2}$O on Au(111) for C09$_{x}$-vdW (left side) and BEEF-vdW (right side) exchange-correlation functionals

The stablest structures with coverage $\theta$=2/9 ML, obtained by C09$_{x}$-vdW and BEEF-vdW exchange-correlation functionals, are presented in Fig. 3. According to the methodology involved in this article, the adsorption energy per molecule for C09$_{x}$-vdW is E$_{ads}=-2.34$ eV, and for BEEF-vdW E$_{ads}=-1.36$ eV. These values indicate that both processes are thermodynamically favorable. These adsorption energies predict the formation of monolayers of HOCH$_{2}$CH$_{2}$O on the gold surface. Besides, critical points and bond paths for these systems are depicted in Fig. 4. Similarly to ethylene glycol on Au(111), we found non-covalent interactions O$\cdot \cdot \cdot$Au and H$\cdot \cdot \cdot$Au. Bond distances, electron density at bond critical points, and its Laplacian are reported in Table 3. Bond distances between oxygen atoms from the HOCH$_{2}$CH$_{2}$O molecule and the Au(111) surface, O$\cdot \cdot \cdot$Au, are within the range 2.22$-$2.81 Å, which is characteristic of non-covalent interactions. For the H$\cdot \cdot \cdot$Au contact, the distance is in the range 2.19$-$3.02 Å. As we mentioned above, the H$\cdot \cdot \cdot$Au contact cannot be overlooked. These results are in good agreement with their respective $\rho _{BCP}$ and $\nabla ^{2}\rho _{BCP}$ values. Also, for this case, the C09$_{x}$-vdW exchange-correlation functional predicts more interactions with shorter distances than BEEF-vdW.

**Fig. 6:**
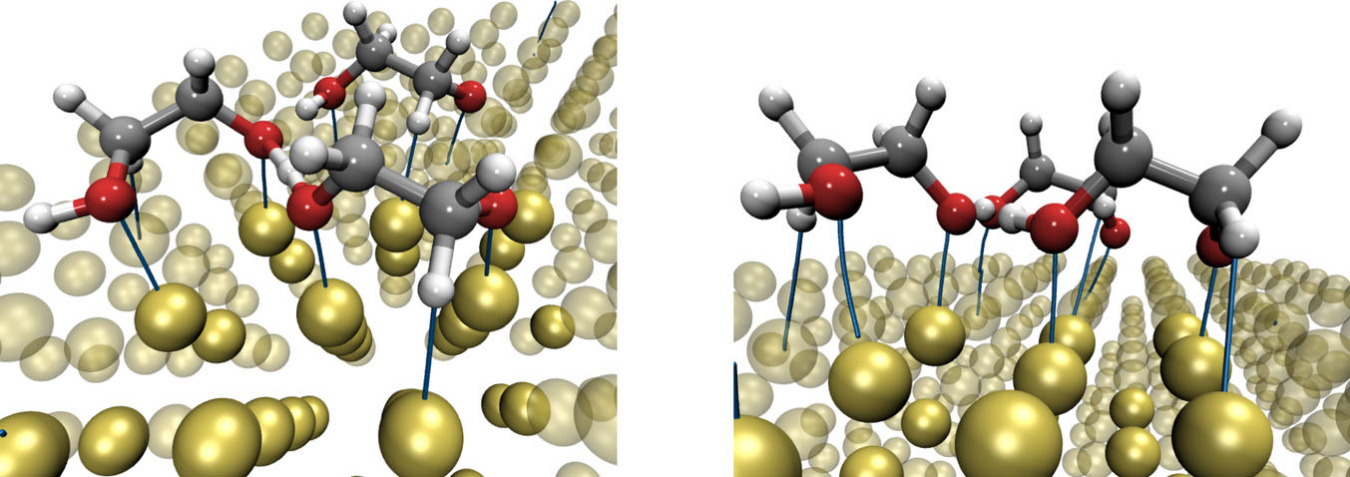
Bond paths for $\theta$=3/9 ML of HOCH$_{2}$CH$_{2}$O on Au(111) for C09$_{x}$-vdW (left side) and BEEF-vdW (right side) exchange-correlation functionals

**Table 4:** Contacts; bond distances, d$_{A-B}$; electronic density in the bond critical point, $\rho _{BCP}$; and Laplacian of the density in the BCP, $\nabla ^{2}\rho _{BCP}$ for three molecules of HOCH$_{2}$CH$_{2}$O adsorb on Au(111) with C09$_{x}$-vdW and BEEF-vdW for $\theta$=3/9 ML

Interaction	d$_{A-B}$ (Å)	$\rho _{BCP}$ (u.a.)	$\nabla ^{2}\rho _{BCP}$ (u.a.)
C09$_{x}$-vdW			
O$\cdot \cdot \cdot$Au	2.15	0.094	0.136
O$\cdot \cdot \cdot$Au	2.20	0.085	0.238
O$\cdot \cdot \cdot$Au	2.29	0.068	0.133
O$\cdot \cdot \cdot$Au	2.34	0.062	0.023
O$\cdot \cdot \cdot$Au	2.35	0.061	0.159
O$\cdot \cdot \cdot$Au	2.49	0.046	0.136
H$\cdot \cdot \cdot$Au	2.19	0.036	0.076
H$\cdot \cdot \cdot$Au	2.42	0.024	0.048
H$\cdot \cdot \cdot$Au	2.34	0.027	0.054
H-O	1.04	0.282	$-$1.314
H-O	1.04	0.281	$-$1.145
H-O	1.05	0.275	$-$1.070
H$\cdot \cdot \cdot$O	1.48	0.088	$-$0.016
H$\cdot \cdot \cdot$O	1.52	0.079	$-$0.263
H$\cdot \cdot \cdot$O	1.52	0.075	$-$0.005
BEEF-vdW			
O$\cdot \cdot \cdot$Au	2.20	0.082	0.089
O$\cdot \cdot \cdot$Au	2.22	0.080	0.225
O$\cdot \cdot \cdot$Au	2.41	0.054	0.081
O$\cdot \cdot \cdot$Au	2.42	0.048	0.154
O$\cdot \cdot \cdot$Au	2.59	0.033	0.064
O$\cdot \cdot \cdot$Au	2.77	0.024	0.057
H$\cdot \cdot \cdot$Au	2.50	0.018	0.043
H$\cdot \cdot \cdot$Au	2.65	0.014	0.033
H$\cdot \cdot \cdot$Au	3.00	0.007	0.021
H-O	1.00	0.350	$-$4.905
H-O	1.01	0.347	$-$2.988
H-O	1.02	0.306	$-$1.817
H$\cdot \cdot \cdot$O	1.56	0.071	0.030
H$\cdot \cdot \cdot$O	1.63	0.060	0.041
H$\cdot \cdot \cdot$O	1.66	0.054	0.064

A new interaction was found between HOCH$_{2}$CH$_{2}$O molecules. In Fig. 4, it is evident a -O$\cdot \cdot \cdot$H$\cdot \cdot \cdot$O- group. Such a behavior is predicted by the two vdW-DFT exchange-correlation functionals considered in this article. The values of $\rho _{BCP}$ and $\nabla ^{2}\rho _{BCP}$ for these interactions are reported in Table 3. According to the sign of the Laplacian of the electron density evaluated at this critical point, both interactions are classified as covalent interactions. However, distances and $\rho _{BCP}$ values suggest that one of these contacts is not necessarily a covalent bond. This contact is within the boundaries between covalent and non-covalent since $\rho _{BCP}$ values in Table 3 are not common for non-covalent interactions.

The interaction between HOCH$_{2}$CH$_{2}$O molecules on the Au(111) surface motivated to increase the cover on the same surface. The highest number of HOCH$_{2}$CH$_{2}$O molecules on the Au(111) surface tested in this article was the adsorption of three HOCH$_{2}$CH$_{2}$O molecules on the Au(111) surface, which corresponds to $\theta$=3/9 ML. The optimized structures shown in Fig. 5 represent the stablest structures found by C09$_{x}$-vdW and BEEF-vdW exchange-correlation functionals; the corresponding adsorption energies per molecule are $-$2.35 and $-$1.35 eV, respectively. Thus, for both covers, $\theta$=2/9 and $\theta$=3/9, C09$_{x}$-vdW and BEEF-vdW exchange-correlation functionals predict strong binding energies in the formation of monolayers of HOCH$_{2}$CH$_{2}$O on the Au(111) surface. The coverage does not influence the binding energy per molecule. Thus, the greater the coverage, the higher the total binding energy. This indicates that no cooperative effects are present in the system, as the Au-O interactions significantly contribute to the total binding energy.

Besides, the interactions between the 3 HOCH$_{2}$CH$_{2}$O molecules and the Au(111) surface were determined and classified using QTAIM (Fig. 6); such results for $\theta$=3/9 ML are contained in Table 4. From this table, it is clear that O$\cdot \cdot \cdot$Au interactions are similar for both covers. However, the number of H$\cdot \cdot \cdot$Au contact is increased. In addition, the H$\cdot \cdot \cdot$O contact between molecules is also present, similar to the results found for $\theta$=2/9 ML. Thus, all contacts found between Au and HOCH$_{2}$CH$_{2}$O molecules are non-covalent. However, the O$\cdot \cdot \cdot$Au link exhibits the highest value of $\rho _{BCP}$, suggesting an electrostatic contribution for this contact, and for O$\cdot \cdot \cdot$Au interaction, dispersion forces command the contact.

In summary, results obtained by C09$_{x}$-vdW and BEEF-vdW exchange-correlation functionals predict monolayers of HOCH$_{2}$CH$_{2}$O molecules on the Au(111) surface, where oxygen and hydrogen atoms interact with gold atoms. The formation of -O-H-O- links between HOCH$_{2}$CH$_{2}$O molecules is an important result delivered by these exchange-correlation functionals.

**Fig. 7:**
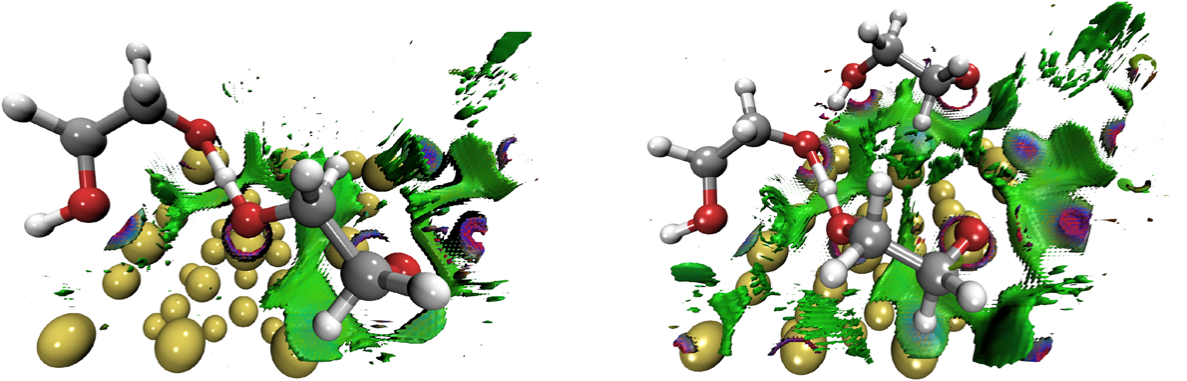
Top view of non-covalent interactions analysis for different cover ($\theta$=2/9 ML left, $\theta$=3/9 ML right) of HOCH$_{2}$CH$_{2}$O with C09$_{x}$-vdW, *s*=0.5 u.a

A non-covalent interactions (NCI) index analysis was performed for both covers, $\theta$=2/9 and $\theta$=3/9, of HOCH${_2}$CH${_2}$O over the Au(111) surface. Results of this scalar field are presented in Fig. 7, which correspond to results found by the C09$_{x}$-vdW exchange-correlation functional. Similar results are found by the BEEF-vdW exchange-correlation functional. In this case, a top view is depicted in Fig. 7 where green isosurface represents weak attractive interactions. The isosurface presents blue tones for the contacts found by NCI, indicating strong non-covalent interactions. In addition, large green zones appear for the highest cover, which suggests an increment of vdW interactions when the number of molecules adsorbed on the gold surface is increased.

## Conclusions

Two exchange-correlation functionals designed to take into account dispersion contributions were used to describe the interaction between ethylene glycol and the Au(111) surface. Previous studies reported interactions between the oxygen atoms of the ethylene glycol and Au atoms. However, for the first time, there is evidence that hydrogen atoms are also involved in the stabilization of these systems. Quantum theory of atoms in molecules results show that H$\cdot \cdot \cdot$Au contacts cannot be overlooked since they have an important role; they are as important as H$\cdot \cdot \cdot$Au contacts. This article also studied the dehydrogenated ethylene glycol on the Au(111) surface. Contacts similar to those of the previous system were observed in this system. However, interactions between the molecules of the dehydrogenated ethylene glycol occur through -O-H-O-links, forming chains. Such a link is interesting because the hydrogen atom forms strong contacts with the involved oxygen atoms. These contacts were unexpected, and for this reason, our group is exploring similar systems to obtain general conclusions.

## Data Availability

No datasets were generated or analyzed during the current study.
